# Supplementary material for: Methanogenic Archaea in the Pediatric Inflammatory Bowel Disease in Relation to Disease Type and Activity
Source: Int J Mol Sci. 2024 Jan 4;25(1):673. doi: 10.3390/ijms25010673 (PMC10779203; doi:10.3390/ijms25010673)
Supplement: Supplementary file 1 [file ijms-25-00673-s001.zip › ijms-2776503-supplementary.pdf]

Table S1. Detailed diagnoses of the patients from the control group.

Diagnosis	No. of patients
Hirschsprung's disease	1
Functional intestinal disorders, unspecified	2
Feeding difficulties and incorrect feeding	1
Gastroesophageal reflux with esophagitis	1
Gastroesophageal reflux	2
Pain in the abdominal and pelvic area	1
Streptococcal pharyngitis	1
Unspecified arthritis	1
Pain located in the lower abdomen	1
Fructose metabolism disorders	2
Other laryngeal diseases	1
Different and unspecified abdominal pain	1
Paralytic syndrome, unspecified	1
Other specified metabolic disorders	1
Chronic mucous otitis media	1
Different and unspecified abdominal pain	1
Other signs and symptoms affecting the gastrointestinal tract and abdominal cavity	1
Other ear disorders in diseases classified elsewhere	1
Embolism and thrombosis of the arteries of the lower limbs	1
Esophageal varices without bleeding	1
Addisonian crisis	1
Other specific diseases of the digestive system	1
Dwarfism	1
Food allergies	1
